# ranacapa: An R package and Shiny web app to explore environmental DNA data with exploratory statistics and interactive visualizations

**DOI:** 10.12688/f1000research.16680.1

**Published:** 2018-11-01

**Authors:** Gaurav S. Kandlikar, Zachary J. Gold, Madeline C. Cowen, Rachel S. Meyer, Amanda C. Freise, Nathan J.B. Kraft, Jordan Moberg-Parker, Joshua Sprague, David J. Kushner, Emily E. Curd

**Affiliations:** 1 Department of Ecology and Evolutionary Biology, University of California, Los Angeles, Los Angeles, CA, 90095, USA; 2 Department of Microbiology and Microbial Genetics, University of California, Los Angeles, Los Angeles, CA, 90095, USA; 3 Channel Islands National Park, National Park Service, Ventura, CA, USA

**Keywords:** environmental DNA, data visualization, citizen science, community science, shiny, metabarcoding, education, community ecology

## Abstract

Environmental DNA (eDNA) metabarcoding is becoming a core tool in ecology and conservation biology, and is being used in a growing number of education, biodiversity monitoring, and public outreach programs in which professional research scientists engage community partners in primary research. Results from eDNA analyses can engage and educate natural resource managers, students, community scientists, and naturalists, but without significant training in bioinformatics, it can be difficult for this diverse audience to interact with eDNA results. Here we present the R package ranacapa, at the core of which is a Shiny web app that helps perform exploratory biodiversity analyses and visualizations of eDNA results. The app requires a taxonomy-by-sample matrix and a simple metadata file with descriptive information about each sample. The app enables users to explore the data with interactive figures and presents results from simple community ecology analyses. We demonstrate the value of ranacapa to two groups of community partners engaging with eDNA metabarcoding results.

## Introduction

The targeted amplification and sequencing of DNA that living organisms shed into their physical environment, termed “environmental DNA (eDNA) metabarcoding,” is revolutionizing microbiology, ecology, and conservation research ( Deiner *et al*., 2017; Taberlet *et al.* 2012). Sequencing of eDNA extracted from field-collected soil, water, or sediment samples can yield insight into a range of questions, from profiling the composition of ancient plant and animal communities ( Pedersen *et al*., 2015), to monitoring populations of rare or endangered species ( Balasingham *et al*., 2018). As the cost of eDNA metabarcoding declines and sample collection techniques become more streamlined (e.g. Thomas *et al*. (2018)), professional research scientists are increasingly using eDNA metabarcoding as a platform to engage a diversity of community partners, including natural resource managers, undergraduate students, and citizen scientists in primary research. However, developing robust and impactful community science programs that engage community partners in all steps of the research process remains a challenge.

eDNA metabarcoding-based projects work well for programs that partner researchers with community scientists because non-experts can be quickly trained to collect samples in the field, and because eDNA metabarcoding is an exciting framework for research pertinent to disciplines such as medicine, agriculture, ecology, and geography ( Deiner *et al*., 2017). Community partners in such programs can have heterogeneous backgrounds, ranging from curious members of the public for whom collecting samples in the field is their first scientific research experience (e.g. University of California’s CALeDNA program), to professional natural resource managers who regularly collaborate with research scientists (e.g. Center for Ocean Solutions’ eDNA project). A key ingredient to promote sustained success of such programs is that community partners should be able to engage across multiple stages of the research project, not only in sample collection ( European Citizen Science Association, 2015; Pandya, 2012). This can be a challenge for community science programs because although it is relatively easy to train community partners to collect eDNA samples, it is far more challenging to train them to independently visualize and analyze results from these studies. Indeed, learning the bioinformatic tools necessary for managing the large, multidimensional datasets generated in these studies can be difficult for professional researchers ( Carey & Papin, 2018), let alone for the non-technical audience of some community science programs.

To address this challenge, we created the R package “ *ranacapa*”, at the core of which is a Shiny web app that can be used to visualize results from eDNA sequencing studies and perform simple community ecology analyses. *ranacapa* complements existing visualization platforms (e.g. Phinch ( Bik & Phinch Interactive, 2014), Phyloseq-Shiny ( McMurdie & Holmes, 2015), QIIME2 Viewer), because in addition to interactive visualizations, *ranacapa* includes brief explanations of several core analyses used in eDNA studies and includes links to additional educational resources. *ranacapa* works with community matrices generated via QIIME ( Caporaso *et al*., 2010) or the Anacapa sequence analysis pipeline, the latter being used extensively by the CALeDNA program.

Here, we describe the package and how it is used by two community science partnerships based at the University of California, Los Angeles (UCLA): first, a collaboration between eDNA researchers and resource managers at the National Park Service, and second, a partnership between community ecology researchers and an undergraduate microbiology course at UCLA. As we show in the Use cases, empowering community partners to interact with the data and perform simple but insightful community ecology analyses can help make these collaborations more enriching and valuable to both parties.

## Implementation

At the core of *ranacapa* is a Shiny web app ( Chang *et al*., 2018), which is available at http://gauravsk.shinyapps.io/ranacapa or with *ranacapa::runRanacapa()*. The package also includes two categories of helper functions ( Table 1) that transform user-uploaded taxonomy and metadata tables into *R* objects that can be visualized and analyzed using the Phyloseq ( McMurdie & Holmes, 2013) and Vegan ( Oksanen *et al*., 2018) packages. *ranacapa* is available for installation from Github or CRAN:



                    
                    devtools
                    
                    ::
                    
                    install_github
                    
                    (
                    
                    "gauravsk/ranacapa"
                    
                    )

                    install.packages
                    
                    (
                    
                    "ranacapa"
                    
                    )
                


**Table 1.** Functions included within the ranacapa package.

Name	Description
scrub_seqNum_column	Removes any "xxx_seq_number" columns from the input taxonomy file if present (depends on which version of Anacapa was used to assign taxonomy)
scrub_taxon_paths	Replaces empty cells in input taxonomy tables with “Unknown”
validate_input_files	Verifies that the input taxonomy file and input mapping file meet specifications
convert_biom_to_taxon_table	Converts a phyloseq-imported biom table into an Anacapa-formatted taxonomy table
group_anacapa_by_taxonomy	Summarizes a site-abundance table from the Anacapa pipeline to each unique taxon
categorize_continuous_vector	Categorizes a continuous vector into low, medium, and high
convert_anacapa_to_phyloseq	Converts a site-abundance table from the Anacapa pipeline and the associated metadata file into a phyloseq object
vegan_otu	Creates a community matrix in the vegan package style using a phyloseq object and an otu_table object
custom_rarefaction	Rarefies a phyloseq object to a custom sample depth and with a given number of replicates
pairwise_adonis ^1^	Wrapper function for multilevel pairwise comparison
ggrare ^2^	Makes a rarefaction curve using ggplot2
runRanacapaApp	Runs the ranacapa Shiny app with tabs for interactive visualizations and statistical analyses

^1^ adopted from https://github.com/pmartinezarbizu/pairwiseAdonis (GPL-3 License)

^2 ^adopted from https://github.com/mahendra-mariadassou/phyloseq-extended (GPL-3 License)

**Figure 1.**
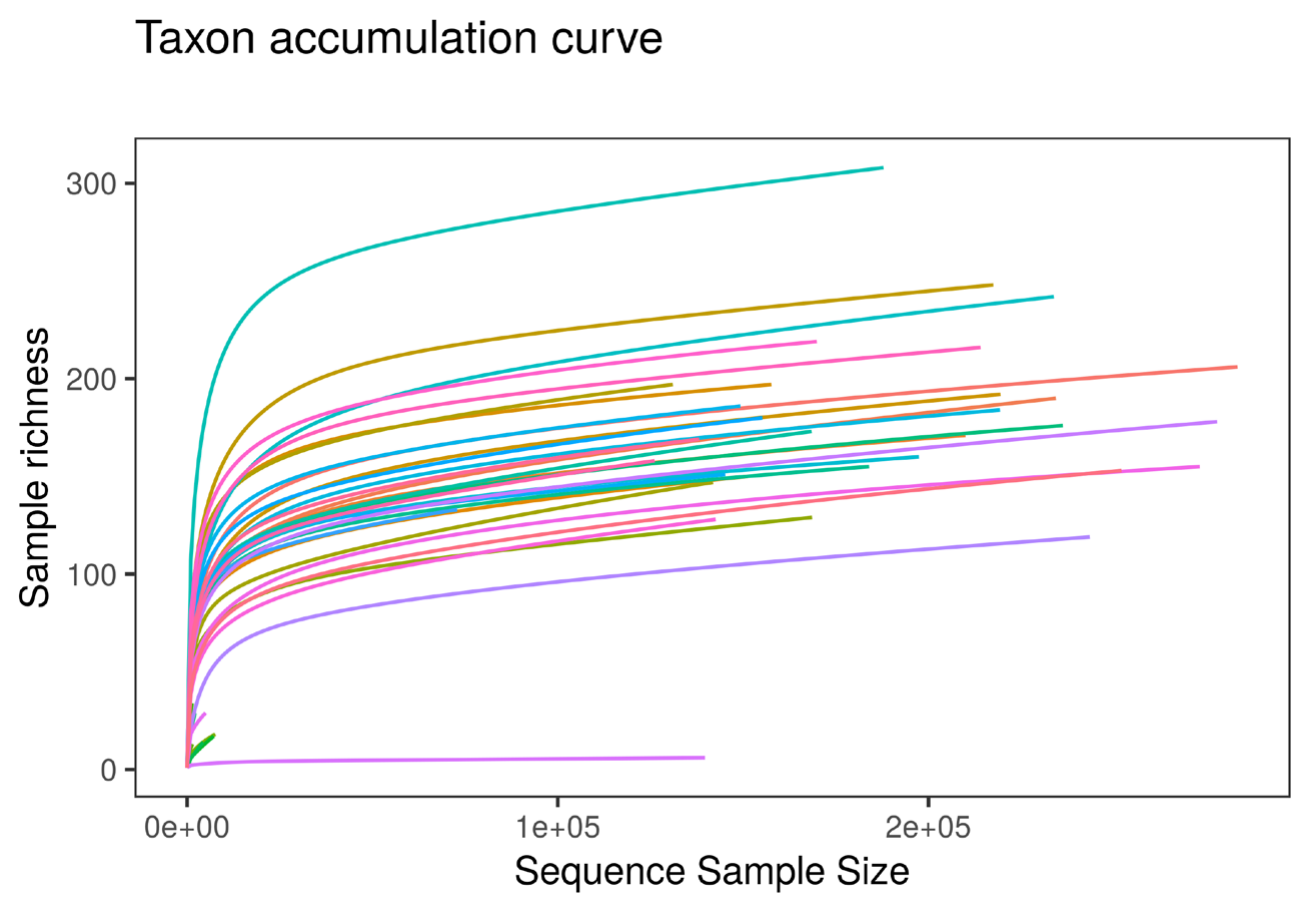
Taxon accumulation curve as shown in the ranacapa Shiny app. The online version of this figure is interactive.

The *ranacapa* Shiny app allows users to interact with eDNA results through statistical summaries and interactive plots, displayed in the following tabs:

**•   Sequencing depth**: Introduces the potential for variation in sequencing depth among samples and explains the basic logic behind rarefying samples in metagenomics studies ( Figure 1). Users can rarefy the dataset to a sampling depth, or can proceed through the rest of the app without rarefying samples. The documentation acknowledges recent disagreement regarding the value of rarefying in metabarcoding and eDNA sequencing studies ( McMurdie & Holmes, 2014).

**Figure 2.**
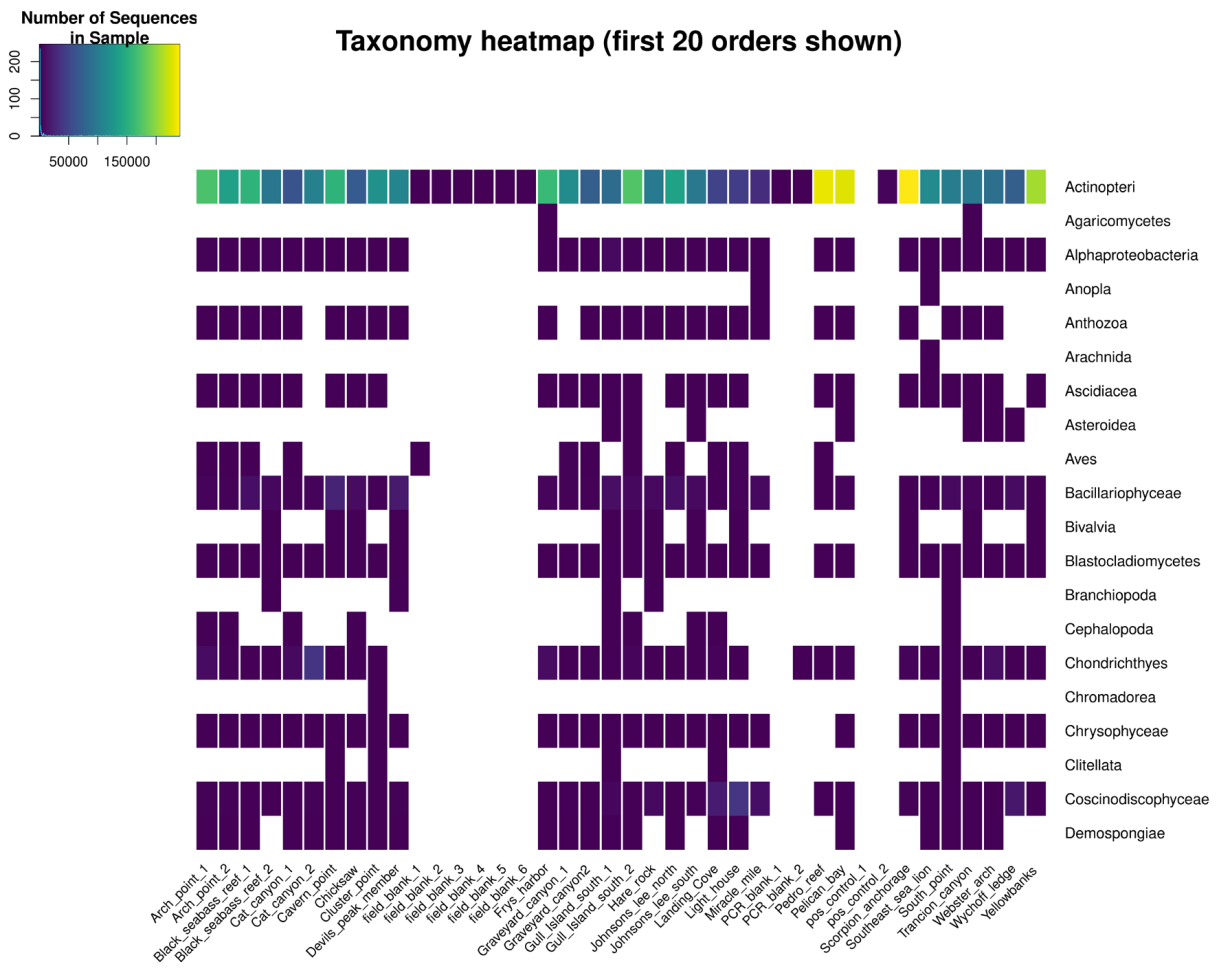
Taxonomy heatmap as shown in the ranacapa Shiny app. Taxonomy is shown at the Order level in this figure; in the app, users can choose the taxonomic level to show in the heatmap. Users can also select or deselect individual taxa to be shown in the heatmap. The online version of this figure is interactive.

**•   Taxonomy heatmap**: Shows the taxon-by-sample matrix as an interactive heatmap made using *heatmaply::heatmaply()* ( Galili *et al*., 2018), where the color of each cell represents the number of times a given taxon was sequenced in a sample ( Figure 2). Users can filter the taxon list by selecting or deselecting specific taxa.

**Figure 3.**
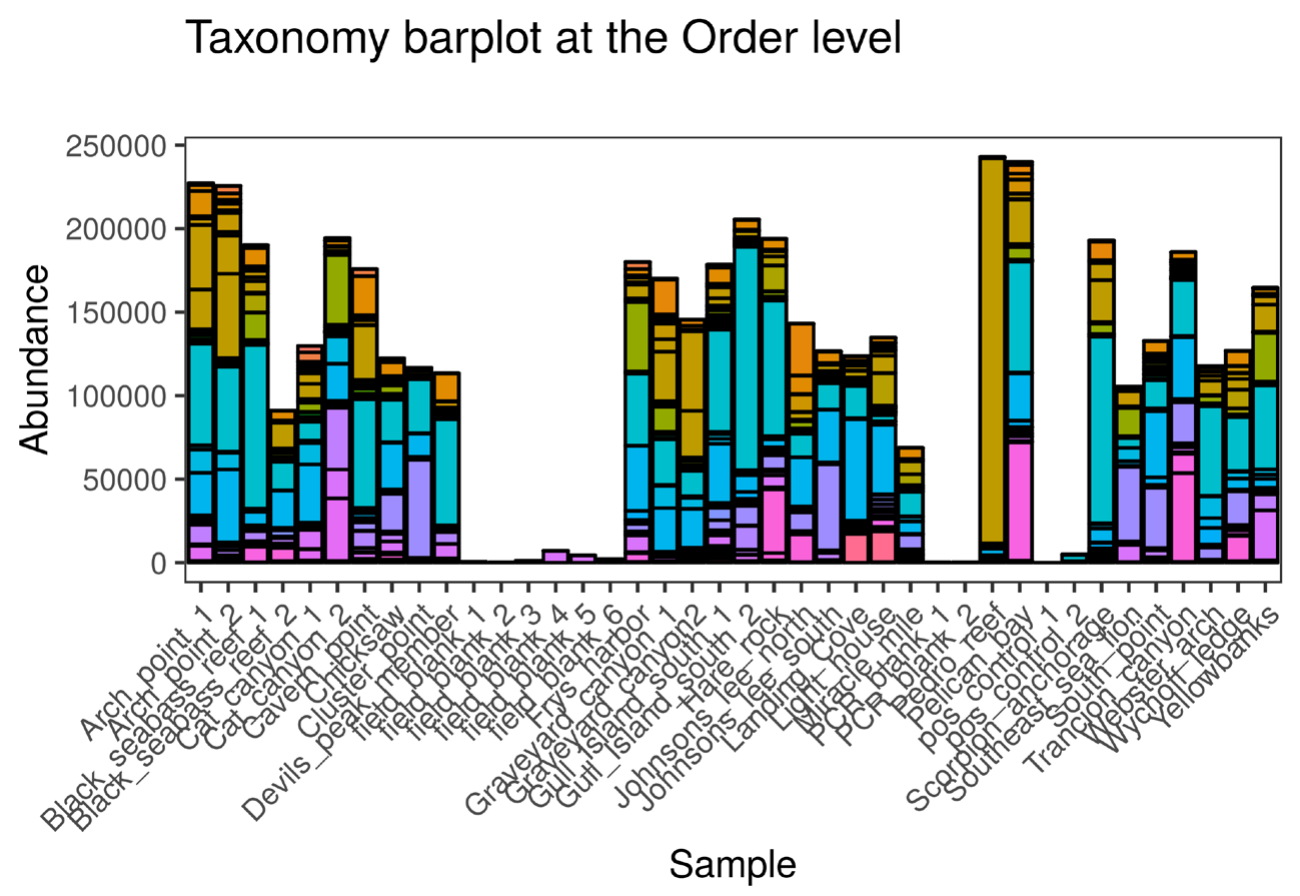
Taxonomy barplot as shown in the ranacapa Shiny app. Taxonomy is shown at the Order level in this figure; in the app, users can choose the taxonomic level to show in the barplot. The online version of this figure is interactive.

**•   Taxonomy barplot**: Shows the taxonomy-by-sample matrix as an interactive barplot ( Figure 3).

**Figure 4.**
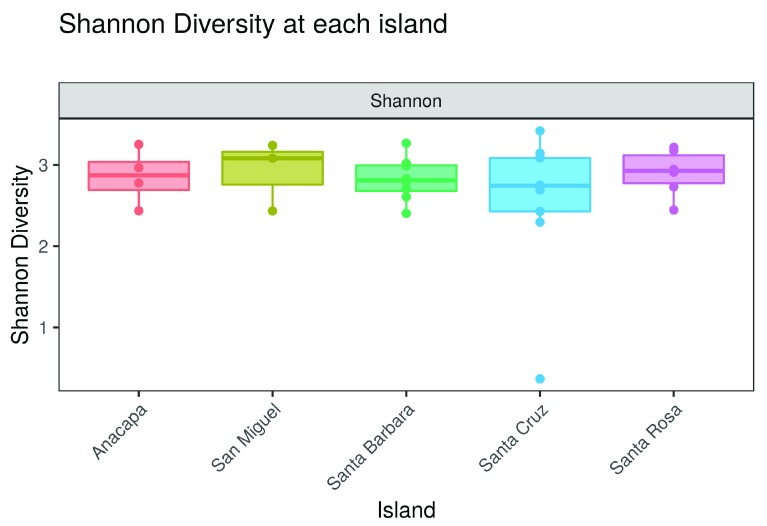
Alpha diversity boxplots as shown in the ranacapa Shiny app. Users can select the X-axis variable using a dropdown menu in the app. The online version of this figure is interactive.

**•   Alpha diversity plots**: Introduces the concept of alpha diversity as the local diversity measured in a single habitat or sample. Users can plot alpha diversity as observed taxon richness or as Shannon diversity per sample, or can group samples according to a variable in the metadata file ( Figure 4).

**•   Alpha diversity statistics**: Allows users to choose a variable from the metadata, and generates an alpha diversity ANOVA table according to the user-selected variable. The tab also shows the output from a post-hoc Tukey test.

**Figure 5.**
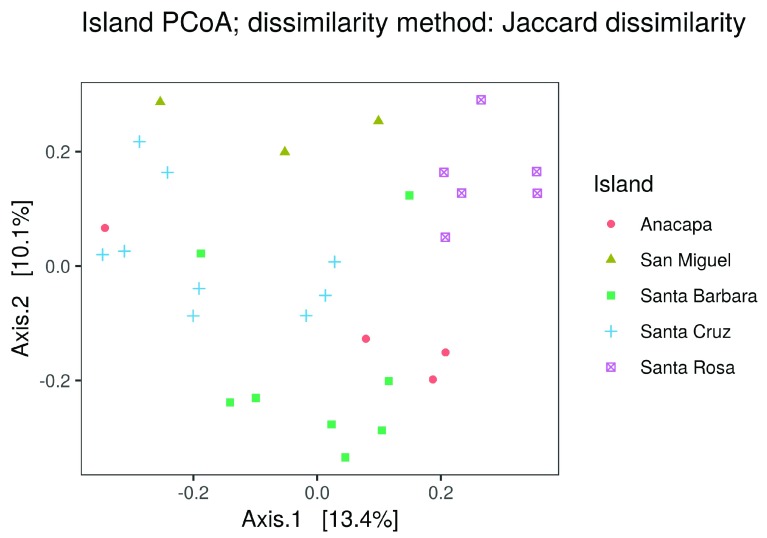
PCoA ordination of the samples as shown in the ranacapa Shiny app. Users can select the grouping variable with a dropdown menu in the app. The online version of this figure is interactive.

**•   Beta diversity plots**: Introduces the concept of beta diversity as the turnover in species composition across habitats (or samples). The tab includes an ordination plot generated by *phyloseq::plot_ordination()*, which in turn uses an ordination object made with *phyloseq::ordinate(., method = "PCoA")*. Points on the PCoA plot are colored according to a user-selected metadata variable ( Figure 5).

    The beta diversity plots tab also includes a dendrogram that groups sites based on Ward’s cluster analysis ( *stats::hclust(distance_object, method = "ward.d2")*), where *distance_object* is made using *phyloseq::distance()*. For both figures, users can toggle between using Jaccard and Bray-Curtis dissimilarity. 

**•   Beta diversity statistics**: Shows results from two statistical tests of species turnover across sites. The first test is a multivariate ANOVA of taxon turnover across sites, implemented with *vegan::adonis()*. The second statistical test, which is implemented with *vegan::betadisper()*, is of heterogeneity of variances among samples. This test compares the degree of sample-to-sample variation within habitats (or within other user-selected groups).

## Operation

*ranacapa* depends on Bioconductor v 3.7, which in turn relies on R v 3.5.0. The Shiny app has been tested on Chrome and Firefox on Windows, Mac-OSX, and Ubuntu.

### Input file structure

The *ranacapa* Shiny app requires two input files. The first requirement is a taxon-by-sample matrix, uploaded either as a rich, dense *.biom* table, or as a tab-separated *.txt* file. Qiime2-generated *.qza* files generated by QIIME2 are not immediately suitable for ranacapa, as they do not contain full taxonomy information. If the site-by-species matrix is uploaded as a *.txt* file, the file should match the specifications of the output files from the Anacapa eDNA sequence analysis pipeline. In Anacapa output, each row represents a taxonomic identification, and each column (save one) represents the number of times that taxon appears in each sequenced sample. One column, named *sum.taxonomy* must contain the taxonomic identification, with taxonomic rank separated by a semicolon, e.g. “ *Chordata;Actinopteri;Chaetodontiformes;Chaetodontidae;Chaetodon;Chaetodon reticulatus* .” A valid input file is structured as follows:



                        
                        sum.taxonomy	Arch_point_1 	Arch_point_2 	Black_seabass_reef_1 Black_seabass_reef_2
<full path>	0		0               0                    0	
<full path>	0		0               43                   87
<full path>	0		0		0		     0
<full path>     0               0               0          	     0
<full path>	24		36              30          	     16
<full path> 	0		0               0                    0
<full path>	0		0               0                    0	
<full path> 	0		0               16                   177
<full path>	0		0               0                    0
<full path>     0		0               0           	     0

                    


The second requirement is a tab-separated *.txt* file that contains sample metadata. The first column in the metadata file should match the sample names in the taxonomy table; the remaining columns contain sample information for each of the samples in the taxon-by-site matrix. The metadata should contain categorical variables with two or more categories per variable. A valid metadata file for the taxonomy table above is structured as follows:



                        
                        Sample 	        	Sample_or_Control  Island	    Protection	  Locality
Black_seabass_reef_1	Sample		   Anacapa          MPA		  Black_seabass_reef
Arch_point_1            Sample 		   Santa Barbara    non-MPA	  Arch_point
Arch_point_2            Sample 		   Santa Barbara    non-MPA	  Arch_point
Black_seabass_reef_2	Sample		   Anacapa          MPA		  Black_seabass_reef
                    


The *ranacapa* function *validate_input_files()* verifies that both the taxonomy table and the metadata files match structural requirements, which are documented in the function help files.

## Use cases

We expect that researchers with expertise in bioinformatics will use the sequence analysis pipeline of their choice to assign taxonomy to eDNA datasets, and generate clean taxonomy and metadata files that can be visualized in *ranacapa*. Researchers can share these files with their partners, and emphasize the analyses or visualizations most appropriate to their use case. We now show how *ranacapa* can facilitate authentic communication between researchers and community partners in two settings.

### Use case 1: Partnership between eDNA researchers and natural resource managers

A team of UCLA researchers partnered with resource managers at the Channel Islands National Park Service to assess the potential for eDNA as a biodiversity monitoring tool to supplement time-intensive visual biodiversity surveys in the Southern California Channel Islands ( Deiner *et al*., 2017; Lessios, 1996; Usseglio, 2015). For this partnership, resource managers collected and filtered 30 unique one-liter water samples for eDNA analysis at permanent monitoring sites inside and adjacent to protected areas, and research scientists at UCLA performed eDNA sequencing of the mitochondrial 12S ( Miya *et al*., 2015) and CO1 ( Leray *et al*., 2013) genes, targeting bony fishes, elasmobranches, and invertebrate taxa. The researchers processed sequences and assigned taxonomy using the Anacapa pipeline, and shared results with the resource managers using the *ranacapa* Shiny app.

The taxonomy heatmap of species detected using the 12S and CO1 metabarcodes ( Figure 2) was the most valuable visualization to this collaboration, because it allowed the resource managers to filter the large observed species list down to a particular set of key taxa that they regularly monitor. The heatmap showed that this pilot study detected 36 of the 70 key metazoans at the species level, and the remaining 34 at the genus, family, or order level. This indicates that eDNA-based studies can provide critical information for ongoing management efforts and provide new insights into the spatial and temporal distributions of these species. The value of *ranacapa* in this scenario was to quickly sort through long species lists generated by eDNA sequencing to highlight the strengths and weaknesses in using eDNA to monitor diversity in the Channel Islands. The data from this study are packaged as the demo dataset for the *ranacapa* Shiny app and are available online ( Kandlikar *et al*., 2018a).

### Use case 2: Partnership between eDNA researchers and an undergraduate microbiology course

A team of community ecology and environmental DNA researchers in the CALeDNA program collaborated with instructors of a research-based environmental microbiology course at UCLA ( Shapiro *et al*., 2015), in which students used eDNA metabarcoding to study the impact of a local wildfire on the plant and soil microbial community. The goal of this twenty-week course was to provide undergraduate students an authentic experience in basic microbiology and microbial community ecology research. Over the first ten weeks, eDNA researchers on the instructional team sequenced the ITS2 ( Gu *et al*., 2013) and 16S SSU RNA ( Caporaso *et al*., 2012) metabarcoding regions from student-collected soil samples and used the Anacapa pipeline to generate taxon-by-sample tables.

The course instructors used the *ranacapa* Shiny app to introduce students to the structure of eDNA sequencing results. The students were encouraged to explore data and perform the statistical analyses most pertinent to the hypotheses they had formed at the beginning of the course. A key benefit of using *ranacapa* was that despite having no prior bioinformatics experience, students could begin exploring the biodiversity in their samples in a matter of minutes by using the online instance of the Shiny app. This allowed the instructors to focus classroom time on biological questions rather than on troubleshooting bioinformatics problems, as had been the case in previous sessions of the course. The course instructors noted that visualizing eDNA data in *ranacapa* helped students understand the relationships between taxon-by-site matrices and the various metadata they had collected in the field. By significantly reducing the time and difficulty in visualizing basic biodiversity patterns, *ranacapa* helped students develop and pursue more sophisticated analyses during the remainder of the course, using tools such as STAMP ( Parks *et al*., 2014) and PICRUSt ( Langille *et al*., 2013). The taxonomy tables and metadata files used in this course are available online ( Kandlikar *et al*., 2018b).

## Summary and future directions

Metabarcode sequencing of environmental DNA is becoming a key tool in a wide variety of ecological studies, and results from these studies are of interest to a broad audience. Our *R* package and Shiny app *ranacapa* helps users conduct exploratory analyses and visualizations on eDNA datasets, and is a step toward more fully engaging participants in all phases of eDNA sequencing-based community science projects.

We propose three avenues for future work with ranacapa. First, we plan to use *ranacapa* as the primary tool to present eDNA results from hundreds of samples sequenced by the CALeDNA community science program. Second, *ranacapa* is being integrated into the upcoming undergraduate curriculum module “Pipeline for Undergraduate Microbiome Analysis”, which will be an open-source, comprehensive suite of analysis and data visualization tools for undergraduate researchers. Finally, in the long-term, we believe there is great promise in linking *ranacapa* with packages that connect with APIs of online biodiversity databases (e.g. Taxize ( Chamberlain & Szöcs, 2013), rinat ( Barve & Hart, 2017)). This will help users explore a much wider range of biodiversity questions, for example, by programmatically asking whether their samples include invasive species that are absent from other nearby sites. In sum, tools like *ranacapa* that allow non-technical audiences to easily interact with results from eDNA sequencing studies have great potential to engage community partners with a wide range of backgrounds and interests in primary research.

## Software availability

•   A Shiny app, including a dataset generated for demonstrations, is available at https://gauravsk.shinyapps.io/ranacapa

• Source code is available from GitHub: https://github.com/gauravsk/ranacapa

•   Archived source code at time of publication: http://dx.doi.org/10.5281/zenodo.1464285 ( Kandlikar & Cowen, 2018)

•   Software license (GPL-3)

## Data availability

Datasets used for the Use cases are available from Figshare:

Dataset 1: Taxon table and metadata file for Channel Islands eDNA samples (mitochondrial 12S and CO1 metabarcodes sequenced) https://doi.org/10.6084/m9.figshare.7199477.v1 ( Kandlikar *et al*., 2018a)

Dataset 2: Taxon table and metadata file for Santa Monica Mountains eDNA samples (16S and plant-ITS metabarcodes sequenced) https://doi.org/10.6084/m9.figshare.7199510.v1 ( Kandlikar *et al*., 2018b)

Both datasets are available under a CC-BY 4.0 license
