# Supplementary material for: ranacapa: An R package and Shiny web app to explore environmental DNA data with exploratory statistics and interactive visualizations
Source: F1000Res. 2018 Nov 1;7:1734. [Version 1] doi: 10.12688/f1000research.16680.1 (PMC6305237; doi:10.12688/f1000research.16680.1)
Supplement: Peer review history

# Peer review: Referee response for version 1

**DOI:** 10.5256/f1000research.18233.r40128

**Published:** 2018-12-19

**Authors:** Niklaus J. Grünwald, Zach Foster

**Affiliations:** 1 Horticultural Crops Research Laboratory, USDA Agricultural Research Service (United States Department of Agriculture), Corvallis, OR, USA; 2 Deptartment of Botany & Plant Pathology, Oregon State University, Corvallis, OR, USA

The authors present the R package rancapa that implements an interactive shiny web app available at https://gauravsk.shinyapps.io/ranacapa for exploration of environmental DNA characterized by amplicon sequencing. This app facilitates many of the standard tests and plots done for metabarcoding data. The app requires two dataset to run, namely a taxonomy-by-sample matrix file and a simple metadata file with descriptive information on the actual environmental samples. This package is particularly useful to anybody interested in exploring biome diversity but lacking extensive computational and bioinformatics skills. We appreciate the focus on open-source code and accessibility, in particular testing the app with Ubuntu and using the color-blind friendly viridis color palette. There are some minor changes in the style of plots and wording we would like to see, but these are a matter of opinion and do not affect the validity of the results. The authors admirably point out flaws and potential pitfalls in the analysis that often go unmentioned (e.g. the drawbacks of rarefaction). Although there is always room for improvement, we feel this app is quite useful as is and it is described well in the associated article. We suggest many improvements that could be made, but most of these are intended as constructive suggestions.

**Specific comments:**

- keywords: include “R package”

**Introduction**

- The sentence “A key ingredient to promote sustained success of such programs is that community partners should be able to engage across multiple stages of the research project, not only in sample collection” is a bit awkward. Perhaps something like the following would be more clear: “Such programs are more likely to succeed if community partners are able to be involved in multiple stages of the research project, not only sample collection”
- End of paragraph 2: “let alone for the non-technical audience of some community science programs.” We suggest “audience” be replaced with “members”, “partners”, or “collaborators”, since they are involved in the project.

**Implementation**

- Paragraph 1, sentence 1: The command “ranacapa::runRanacapa()” does not work. Should it be “ranacapa::runRanacapaApp()”?
- Figure 4: It would be nice if the results of the Tukey’s HSD test be included on the graph using letter codes.
- Sequencing depth, sentence 2: “Users can rarefy the data set to a sampling depth, or can proceed through the rest of the app without rarefying samples.” It's nice to have the option, but if sequences are not rarefied, perhaps they should be converted to proportions? The taxon abundance plots (e.g. the heat map) is biased without some correction for sampling depth and proportions would avoid the loss of data associated with rarefaction. While converting to proportions will not remove sample-size bias for alpha and beta diversity analyses, it will remove the bias for taxon abundance analyses for the most part. The conversion to proportions could also be an option in just the taxon abundance analyses.
- Beta diversity plots, sentence 1: “Introduces the concept of beta diversity as the turnover in species composition across habitats (or samples).” The word “turnover” seems to imply a change over time, rather than a comparison between communities. Perhaps something like: “Introduces the concept of beta diversity as a way to compare community composition across habitats (or samples).”
- Beta diversity statistics, sentence 1: “Shows results from two statistical tests of species turnover across sites.” See comment above on “turnover”.

**Suggestions on the software**

- Exploring ranacapa at https://gauravsk.shinyapps.io/ranacapa shows the interactive tools. We suggest that screenshots from the actual shinyapp be presented as figures as they provide the context for exploring the data. For example under Figure 2, the legend states that users can also select or deselect individual taxa’ but the figure 2 in the manuscript does not show this. This is confusing.
- We also suggest giving explicit examples for executing the shiny app on a desktop maybe right after the installation instructions.

**Data import**

- The proper format for input data is described in the article, but it would be nice if it was repeated in the “data import” section of the app as well. You could also include tips to convert common formats to the needed format here.

**Sequencing depth**

- Second paragraph, first sentence: “In eDNA sequencing, such variation in how deeply a given sample is sequenced can happen for a variety for reasons-”. Should the “-” be a “:”?
- Third paragraph, first sentence: You use double quotes for “deeply” in the first paragraph, but single quotes for ‘rarefy’.
- Add proportions as an alternative to rarefaction instead of “none”?
- The rarefied samples plot is a good idea, but hard to read in its current state. Perhaps change the faceting settings so that there is one column of plots or have a option to choose the sample shown?
- Taxonomy bar pot: The number of similar colors make this hard to read without selecting individual taxa. We know this is a limitation of stacked barcharts, but it might make it more readable to sort the taxa by average abundance and then stagger the colors like so: For example, if there are 30 taxa, then the first 10 get colors 1, 4, 7, 10, 13, 16, 19, 22, 25, 28 (seq(1,30, 30 / 10)). Also have the most abundant taxa be the lowest on the stacked bars. That way adjacent colors can be differentiated and the colors of rare taxa won't be confused with the colors of the abundant taxa.

**Taxonomy heatmap**

- How about faceting by sample metadata or clustering samples by similarity? You could use the results of the Ward’s Hierarchical Clustering like you do in the beta diversity section.
- Can you make the height of the plot depend on the number of samples plotted? Also, making the “Select taxa to visualize” box taller would be nice.

**Alpha diversity plots**

- You use the term “species richness” without explicitly defining it, although you implicitly define it. It might be a good idea to point out that “observed” is the same as “species richness”.
- Plotting the results of the Tukey’s HSD test on this plot would be great. Actually this tab could probably be combined with the “alpha diversity stats” tab.
- The color and the different point shapes are not needed, but that’s just a question of style.

**Alpha diversity stats**

- You say “ANOVAs only work when each group is represented by a few samples”, which is great to point out. How about just not allowing for the ANOVA and Tukey’s HSD if there are not multiple samples per treatment?
- How about not showing the Tukey’s test result if the ANOVA p-value is more than 0.1? You do say “the following results are meaningful only if the ANOVA table above suggests that sites are in fact unequal in terms of their diversity”, but it's very tempting to look at the results of the Tukey’s HSD test anyway. Alternatively, you could add a warning above the Tukey’s HSD results if the ANOVA p-value is more than 0.1.
- How about using the DT package to make the Tukey’s HSD results table sortable, like you do for other tables? The first thing most people will want to do is sort by p-value.

**Beta diversity plots**

- You say “samples that are distant in this plot have very different species lists”. That is not exactly true. Samples that are more distant from each other are more different from each other, but they all might be quite similar in terms of the species present; The PCoA will always show differences, no matter how small.
- An explanation of how to interpret the percent of the variation explained by each axis would be nice.
- How about confidence interval ellipsis for the sample types?
- How about allowing exploration of other dimensions (maybe in a 3d plot as well)?
- In the clustering dendrogram, how about coloring sample names by sample type? Ideally, it would be the same colors used in the PCoA plot, so you would not need a second legend.

**Beta diversity stats**

- How about displaying the results of the Adonis analysis in a table instead of a printed data.frame, like how it is done in the “Homogeneity of Variances” analysis? Tables from the DT package would be nice, so we can sort by p-value.

**Data export**

- It would be nice to provide an option to export the data in the taxmap format from the taxa package ^1^. Zach Foster can help implement this.

We have read this submission. We believe that we have an appropriate level of expertise to confirm that it is of an acceptable scientific standard.

## References

[1] FosterZSLChamberlainSGrünwaldNJ: Taxa: An R package implementing data standards and methods for taxonomic data. *F1000Res*.2018;7: 10.12688/f1000research.14013.2 272 10.12688/f1000research.14013.2 29707201PMC5887078

# Peer review: Referee response for version 1

**DOI:** 10.5256/f1000research.18233.r40129

**Published:** 2018-12-14

**Authors:** Holly M. Bik

**Affiliations:** 1 Department of Nematology, University of California, Riverside, Riverside, CA, USA

This article presents a new R software package and shiny app called "ranacapa", aimed at producing rapid visualization and exploration of eDNA metabarcoding datasets. Overall I found this software tool to be exciting and user friendly, and the outlined use cases provided good examples of the need for such tools amongst end users including researchers and undergraduate students. The R shiny app was easy to explore using the demo dataset, and the plots were responsive and visually useful. 

 When trying to install ranacapa in RStudio (via install.packages), I am consistently getting an error message stating that "package ‘ranacapa’ is not available (for R version 3.3.2)". Furthermore, ranacapa does not appear to be listed as a package currently available in CRAN

 (https://cran.r-project.org/web/packages/available_packages_by_name.html#available-packages-R).

 Further investigation suggests that this package requires R 3.5.0 or higher to run, but this is not obvious for end users with older versions of R installed. Is it possible to add back compatibility to older versions of R? Especially for end users using HPC facilities who may be unable to update the base R version on their cluster.

 "Taxonomy-by-sample matrix" - since ranacapa uses a non-standard input (essentially an OTU table summarized by taxonomy), it would be useful to provide specific instructions and/or a conversion script where users can take their QIIME BIOM files or tab-delimited OTU tables and convert them into a format that is suitable for uploading into ranacapa. Users with minimal technical expertise may struggle to convert their data in the correct way. Especially useful would be R scripts or package commands (e.g. phyloseq) that will output taxonomy-by-sample-matrixes in the correct format.

 The R shiny web app does not provide much information regarding file formatting (users must read the F1000 article for specific information). The only documentation online states that the imported files should be in .biom or .txt formats. What format(s) are supported for the header row (commented out vs. plain text)? Are there any limitations on sparse vs. dense or JSON vs. HDF5 BIOM files (e.g. from QIIME 1 or QIIME2)? Detailed information and documentation should be provided on the website itself in addition to the F1000 article.

 For the metdatata file - can users directly import QIIME-formatted mapping files (e.g. with a header row beginning with #SampleID that has been validated via Keemi)? Or does this file need to be be edited further for ranacapa?

 For the demo file on the web server - what study did this file come from? It would be useful to have some contextual information about the file, eDNA markers used, questions/hypotheses, and study design so that end users can explore the demo dataset in a more thoughtful way. 

 Some components of the R Shiny web app do not always seem to function correctly over a slow internet connection - it seems to freeze up and require re-loading, and/or users are presented with an error message. This error message sometimes goes away and the plots pop up as expected after a few minutes. Better error dialogs or "please wait" message boxes would be much less frustrating for the end user to figure out what is happening.

 Sequencing Depth - the rarefaction depth slider bar is straightforward and should be familiar to most users, however, what impact do multiple rarefactions have on the displayed graphs? Are the rarefaction curves averaged somehow before being displayed? Further information on this parameter would be useful to include in the explanatory text.

 Taxonomy heatmap - when choosing a lower taxonomic level, the taxonomy strings are overlapping and unreadable. Zooming in cuts off some of the categories on the x-axis. It would be better to be able to expand the heatmap along the y-axis (stretch it out) so you can read the taxonomy labels while still being able to view all of the heatmap information.

 Taxonomy heatmap - when selecting a taxon to visualize, users might want to see more detail at lower taxonomic levels, however, selecting a taxon to visualize and then choosing a different taxonomic rank currently removes this filter choice and reverts back to showing all taxa at that level.

 Alpha Diversity Plots - what is the difference between Observed vs. Shannon diversity visualizations? The explanatory text only partially explains the difference between these two options, and could be clarified to explicitly state the important differences in these two calculations. Furthermore, what do high vs. low numerical values mean for each of the two indices, in biological terms (e.g. lower number of species)? These kind of diversity statistics may be familiar for ecologists and microbiome researchers, but are likely to be confusing for citizen scientists, students, or scientists outside of these fields.

 Alpha Diversity Stats - Unless you have a strong statistical background, it is impossible to interpret the numbers and outputs listed in this tab in the ranacapa shiny app. More explanation is needed to guide users in what they should be looking for - what columns are most important in the ANOVA table to see if samples are in fact unequal in terms of their diversity? In the post-hoc tests, should users be looking for P values <0.05 in the last column? 

 Beta Diversity - the Jaccard and Bray-Curtis indexes could be explained more plainly. Jaccard is the simplest measure of shared taxa/OTUs, while Bray-Curtis is an index focused on the most abundant taxa/OTUs in the dataset. Also some explanation of the PCoA axes (e.g. that they explain variation but do not relate to metadata) would be useful. Adding the Canberra index might also be useful here, as this is another useful index which ignores the most abundant taxa and looks at differences across rare species.

 Beta Diversity Stats - same comment as Alpha Diversity stats (see above). Needs more context and explanation to be accessible to end users without a statistical background.

 Use Case 1 - how was the UCLA eDNA dataset filtered down to the set of key taxa that resource managers were looking for? Was this done via R command line, or via the R shiny app interface? Custom filtering is likely to be a common use case, and it would be valuable to add in more specific information regarding how this type of taxon filtering can be accomplished for any dataset.

I have read this submission. I believe that I have an appropriate level of expertise to confirm that it is of an acceptable scientific standard, however I have significant reservations, as outlined above.

# Peer review: Referee response for version 1

**DOI:** 10.5256/f1000research.18233.r40402

**Published:** 2018-12-03

**Authors:** Tristan Cordier

**Affiliations:** 1 Department of Genetics and Evolution, University of Geneva, Geneva, Switzerland

The manuscript “ranacapa: An R package and Shiny web app to explore environmental DNA data with exploratory statistics and interactive visualizations” is describing an R package to visualize metabarcoding data and perform summary statistics. 

 I think that such application will be very useful for quick results sharing within any project that involves stakeholders with various background. Too many projects still rely solely on a printed (or pdf) report with key chosen figures, orienting the readers towards the main messages. I do believe that such application will provide a nice complement to a project report for people that want to dive into the results, or explore the robustness of the conclusion that are highlighted in a report. 

 I also agree with the authors that such tools are particularly interesting for sharing results with non-expert enthusiasts and students. I appreciate the accompanying text to present the specificity of composition (count) data and the possible flaws when analyzing such data. The website “Gusta Me” ( https://mb3is.megx.net/gustame) could maybe be mentioned as an external resource for guiding further the users for the interpretation of results, or to guide any additional analysis. 

 I wonder how such application is intended to be deployed. Do the authors plan to let research scientists deploy the app within their own computational resources or do they plan to make the application accessible ( https://gauravsk.shinyapps.io/ranacapa/) opened to the community? 

 I made few comments on the GUI that the authors may or may not follow. The most important point to address would be to allow users to write their PERMANOVA model. 

 Data import: 

- Maybe add the expected format, with a toy example, indicating the separator character (tab or comma or semi-comma?). This is mentioned in the GitHub page of the project (not the separator though), but I think this would be nice to have it here also. My guess is that many users would not even get to the GitHub page before using the app. 

Sequencing depth: 

- Would it be possible to add a “free” input field? As it is now, it is not easy to get a precise value. The slider does not allow this kind of precision, it increases or decreases by 1000 but on the demo example, it is stuck to 10006. 
- Would it be possible to include normalization as an option? At least the relative abundance for a start, because as you mention on the accompanying text, we are far from having reach a consensus on that matter. 
- What means the first field “select the variable”, do you mean to rarefy not samples but something else? I don’t understand. 

Taxonomy barplot: 

- I personally don’t like taxonomic barplots, but I recognize that they can be useful to easily illustrate some obvious differences (or similarities) between experimental treatments to an audience unfamiliar with metabarcoding data. The lowest taxonomic ranks return a (way) too many categories. In the presented example. Even the highest taxonomic rank (phylum), it yields too many categories to easily visualize what is actually in the samples, because colors are too close to each other. There is no solution, I just don’t like these plots. But it is nice to have this option in the app anyway. 

Taxonomy heatmap:

- Is there an option to scale differently the X and Y axis? In the example dataset, it is actually a similar problem as for taxonomy barplot, lowest taxonomic rank gives too many rows on the plots. A way to better see is to zoom in, but then the horizontal axis is quite messed up. 
- Maybe it would be nice to have input fields to manage those axes and font size. 

Alpha-diversity plots:

- I guess the data behind is the rarefied version of the dataset, but this should be clear. Maybe the box can be there, but in grey to show that it would not make sense to use the raw dataset? 

Alpha-diversity stats: I think this is fine.  

 Beta-diversity plots:

- The users should be able to refine the ordination plots, its size, the size of each axis. 
- Why the ordination plots configuration (and the cluster analysis) is changing when we select different variables to group the dot shapes and colors?  

Beta-diversity analyses:

- Would it be possible to let the user write his own model? As for now, it is not possible to have an interaction term, or a nested model. 
- To be able to select the sample as variable does not make sense. 

I have read this submission. I believe that I have an appropriate level of expertise to confirm that it is of an acceptable scientific standard, however I have significant reservations, as outlined above.
